# Intradermal administration of DNA vaccine targeting Omicron SARS-CoV-2 via pyro-drive jet injector provides the prolonged neutralizing antibody production via germinal center reaction

**DOI:** 10.1038/s41598-023-40172-y

**Published:** 2023-08-10

**Authors:** Hiroki Hayashi, Jiao Sun, Yuka Yanagida, Takako Otera, Jiayu A. Tai, Tomoyuki Nishikawa, Kunihiko Yamashita, Naoki Sakaguchi, Shota Yoshida, Satoshi Baba, Chin Yang Chang, Munehisa Shimamura, Sachiko Okamoto, Yasunori Amaishi, Hideto Chono, Junichi Mineno, Hiromi Rakugi, Ryuichi Morishita, Hironori Nakagami

**Affiliations:** 1 Department of Health Development and Medicine, Osaka University Graduate School of Medicine, Suita, Osaka Japan; 2 Department of Device Application for Molecular Therapeutics, Osaka University Graduate School of Medicine, Suita, Osaka Japan; 3 Department of Geriatric Medicine, Osaka University Graduate School of Medicine, Suita, Osaka Japan; 4 Department of Gene and Stem Cell Regenerative Therapy, Osaka University Graduate School of Medicine, Suita, Osaka Japan; 5 Department of Neurology, Osaka University Graduate School of Medicine, Suita, Osaka Japan; 6 Department of Clinical Gene Therapy, Osaka University Graduate School of Medicine, Suita, Osaka Japan; 7 Division of Microbiology and Immunology, Center for Infectious Disease Education and Research, Osaka University, Suita, Osaka Japan; 8 Anges Inc., Tokyo, Japan; 9 Daicel Co, Osaka, Japan; 10 Takara Bio Inc., Kusatsu, Shiga Japan

**Keywords:** DNA vaccines, Infection

## Abstract

Emerging SARS-CoV-2 Omicron variants are highly contagious with enhanced immune escape mechanisms against the initially approved COVID-19 vaccines. Therefore, we require stable alternative-platform vaccines that confer protection against newer variants of SARS-CoV-2. We designed an Omicron B.1.1.529 specific DNA vaccine using our DNA vaccine platform and evaluated the humoral and cellular immune responses. SD rats intradermally administered with Omicron-specific DNA vaccine via pyro-drive jet injector (PJI) thrice at 2-week intervals elicited high antibody titers against the Omicron subvariants as well as the ancestral strain. Indeed, the Omicron B.1.1.529-specific antibody titer and neutralizing antibody were higher than that of other strains. Longitudinal monitoring indicated that anti-spike (ancestral and Omicron) antibody titers decreased toward 30 weeks after the first vaccination dose. However, neutralization activity remained unaltered. Germinal center formation was histologically detected in lymph nodes in rats immunized with Omicron DNA vaccine. Ancestral spike-specific immune cell response was slightly weaker than Omicron spike-specific response in splenocytes with Omicron-adapted DNA vaccine, evaluated by ELISpot assay. Collectively, our findings suggest that Omicron targeting DNA vaccines via PJI can elicit robust durable antibody production mediated by germinal center reaction against this new variant as well as partially against the spike protein of other SARS-CoV-2 variants.

## Introduction

The development of effective vaccines has been the critical agenda in combating SARS-CoV-2 since the onset of the global COVID-19 (Coronavirus Disease 2019) pandemic^1^. Several companies have produced SARS-CoV-2 vaccines based on new technologies, such as mRNA-based and adenovirus-based vaccines, with a high preventive effect^2–4^. However, the continued evolution of SARS-CoV-2 has generated new variants, resulting in reduced efficiency of the originally approved vaccines or monoclonal antibodies^5–7^. However, since November 2021, the newly emerged Omicron variant (B.1.1.529) has rapidly spread worldwide, with over 30 mutations in the spike protein, leading to robust immune escape mechanisms against neutralizing antibodies in the sera of infected or vaccinated individuals^8–10^. Currently, Omicron variant-adapted mRNA vaccines, including bivalent vaccines against the ancestral strain and the Omicron variant, have been developed and used^11–13^. Despite the high protective effect of mRNA vaccines, one of the limitations is their instability, as they require ultra-cold conditions for storage or transportation^14,15^. Therefore, we require more stable alternative platform vaccines with adequate protection against newer variants of SARS-CoV-2.

As a part of the emerging technologies for vaccine development against COVID-19, nucleic acid-based vaccines have the advantage of rapid development as only genetic information of the virus is required for designing the vaccine^16^. DNA-based vaccines can be stable and exhibit enhanced thermostability than mRNA vaccines^17,18^. The global vaccination might be easier with DNA vaccines for pandemics that may occur in the future. We previously developed a DNA vaccine targeting ancestral SARS-CoV-2, wherein intramuscular or intradermal administration of the DNA vaccine targeting the spike protein could effectively induce neutralizing antibodies and protect hamsters from SARS-CoV-2 infection^19,20^. Moreover, we have improved the DNA vaccine platform for SARS-CoV-2, leading to rapid antibody production compared with that in the original DNA vaccine (GPΔ-DNA vaccine)^21^. Notably, the humoral immune responses generated by intradermal administration of the vaccine via a pyro-drive jet injector were higher than that of intramuscular administration. These results demonstrate that our improved DNA vaccine platform can promptly accommodate emerging SARS-CoV-2 variants with enhanced protective effect^21^.

In this study, we re-designed the Omicron B.1.1.529-targeted DNA vaccine using our developed vaccine platform and evaluated the humoral and cellular immune responses generated against the Omicron sub-variants.

## Results

### Design and in vitro expression of Omicron variant-targeting DNA vaccine

**Figure 1:**
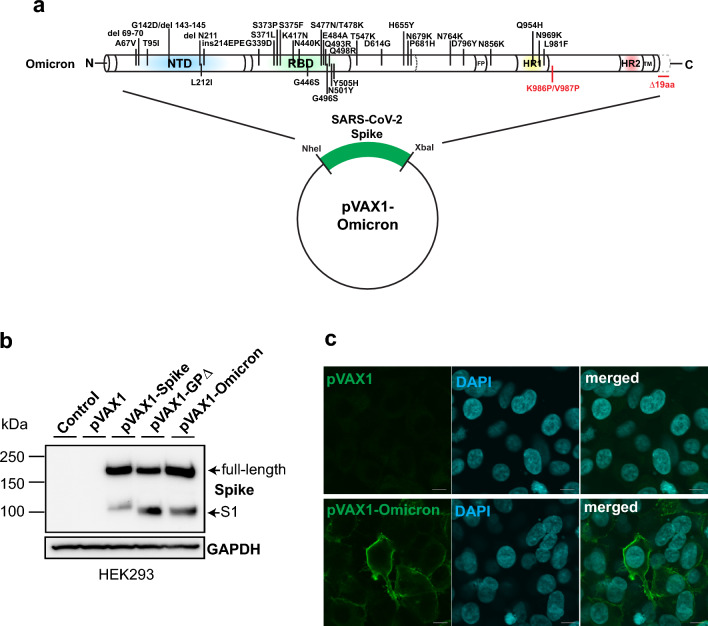
Development of DNA vaccine targeting SARS-CoV-2 Omicron variant. (**a**) Schematic representation of plasmid construction for SARS-CoV-2 Omicron DNA vaccine. K986P/V987P mutation and deletion of 19 aa at the C-terminal were introduced to improve cell-surface antigen expression are highlighted in red. Optimized-Omicron spike sequences were inserted into the pVAX1 vector. (**b**) Expression of Omicron spike induced by Omicron DNA vaccine was detected in HEK293 cells. Upper and lower bands represent the full-length and S1 domain of the spike, respectively. GAPDH was used as a control. The images were cropped from the data shown in Supplementary Fig. 1. (**c**) Cell surface localization of spike protein was evaluated by immunocytochemical analysis in HEK293 cells. pVAX1 control plasmid (upper panels) and Omicron DNA vaccine (lower panels) were transfected into HEK293 cells. Scale bar = 10 μm. See also Supplementary Fig. 1.

We had already developed DNA vaccine platform by additional mutation of K986P/V987P and deletion of 19 amino acids at the C-terminal end, leading to rapid antibody production (GPΔ-DNA vaccine;^21^). Based on this GPΔ-DNA vaccine platform, we re-designed the DNA vaccine for Omicron variant BA.1 by the introduction of mutations and deletions, namely A67V, H69del, V70del, T95I, G142D, V143del, Y144del, Y145del, N211del, L212I, ins214EPE, G339D, S371L, S373P, S375F, K417N, N440K, G446S, S477N, T478K, E484A, Q493R, G496S, Q498R, N501Y, Y505H, T547K, D614G, H655Y, N679K, P681H, N764K, D796Y, N856K, Q954H, N969K, L981F (Fig. 1a). Spike protein expression was evaluated by the transient transfection of HEK293 cells. Omicron spike was induced by the Omicron DNA vaccine (Fig. 1b and Supplementary Fig. 1). The surface expression of the spike protein was monitored (Fig. 1c) and analyzed using the immunocytochemical assay. These data indicated that the spike protein was expressed from the Omicron sequence-matched DNA.

### Omicron DNA vaccine induces humoral immune responses

**Figure 2:**
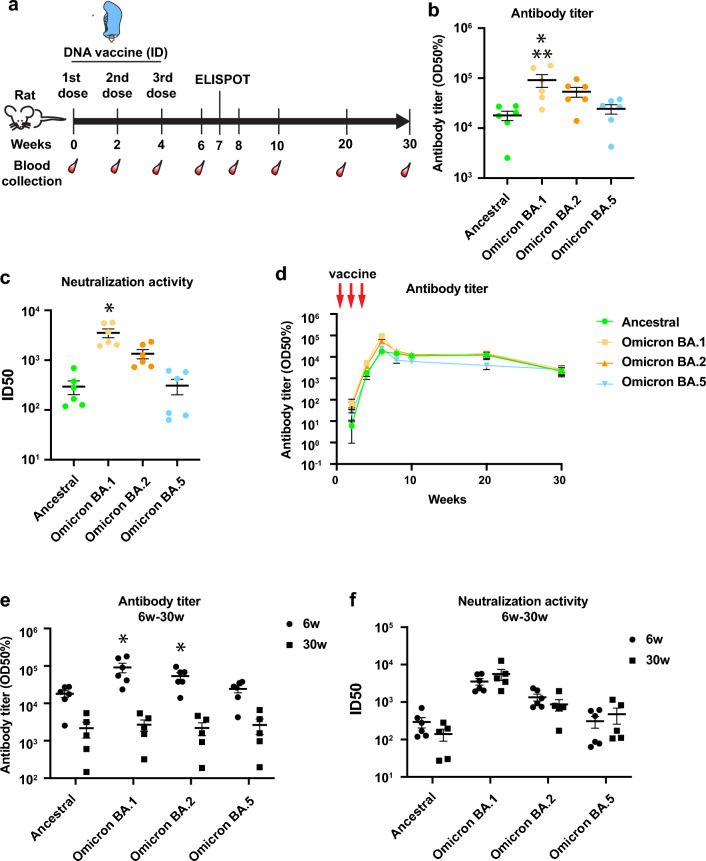
Humoral immune responses were generated by the SARS-CoV-2 Omicron-DNA vaccine. (**a**) Scheme of DNA vaccine administration and blood collection. Female SD rats were intradermally administered with Omicron DNA vaccine via PJI thrice at 2-week intervals (240 μg of DNA vaccine/body). (**b**) Antibody titers for ancestral, Omicron BA.1, BA.2, and BA.5 were measured by ELISA 6 weeks after the first vaccination. *p < 0.01 versus ancestral spike, **p < 0.01 versus BA.5. (**c**) Neutralization activity was measured by pseudo-typed virus against ancestral, Omicron BA.1, BA.2, or BA.5 at 6 weeks after the first vaccination dose. *p < 0.01 versus ancestral, Omicron BA.2, and BA.5, respectively. (**d**) Longitudinal monitoring of antibody titers for ancestral as well as Omicron variants until 30 weeks after the first dose of vaccination. (**e**, **f**) The comparison of the levels of (**e**) antibody and (**f**) neutralization activity between 6 and 30 weeks after the first vaccination dose. *p < 0.05 versus 30 weeks. N = 5–6. Data are presented as mean ± SEM. See also Supplementary Fig. 2–4.

Next, we evaluated whether the Omicron DNA vaccine induced antibody production in vivo. We intradermally administered the Omicron DNA vaccine into SD rats using a pyro-drive jet injector (PJI)^21^ thrice every other week (Fig. 2a) and analyzed antibody titers for ancestral and omicron variants, including BA.1, BA.2, and BA.5. Six weeks after the first vaccination, the antibody titer was increased for all variants; indeed, the antibody titer for BA.1 was the highest, compared with that of the other strains (Fig. 2b and Supplementary Fig. 2). Neutralization activity against BA.1 was also higher than the other variants, analyzed by pseudo-typed virus assay (Fig. 2c). Antibody levels were observed during follow-ups with a high antibody titer being detected even 30 weeks after the first dose of vaccination (Fig. 2d). However, antibody titers for BA.1 and BA.2 were significantly decreased (Fig. 2e). The DNA vaccine-induced IgG subclass were IgG2b dominant, or Th1-based immune response (Supplementary Fig. 3). We also analyzed neutralization activity at 30 weeks using a pseudo-typed virus. Notably, the neutralization activities of all variants remained similar without any decrease (Fig. 2f and Supplementary Fig. 4). These results suggest that the DNA vaccine could induce long-term protection of neutralizing antibody.

### Germinal center formation was detected by PJI-mediated Omicron DNA vaccine

**Figure 3:**
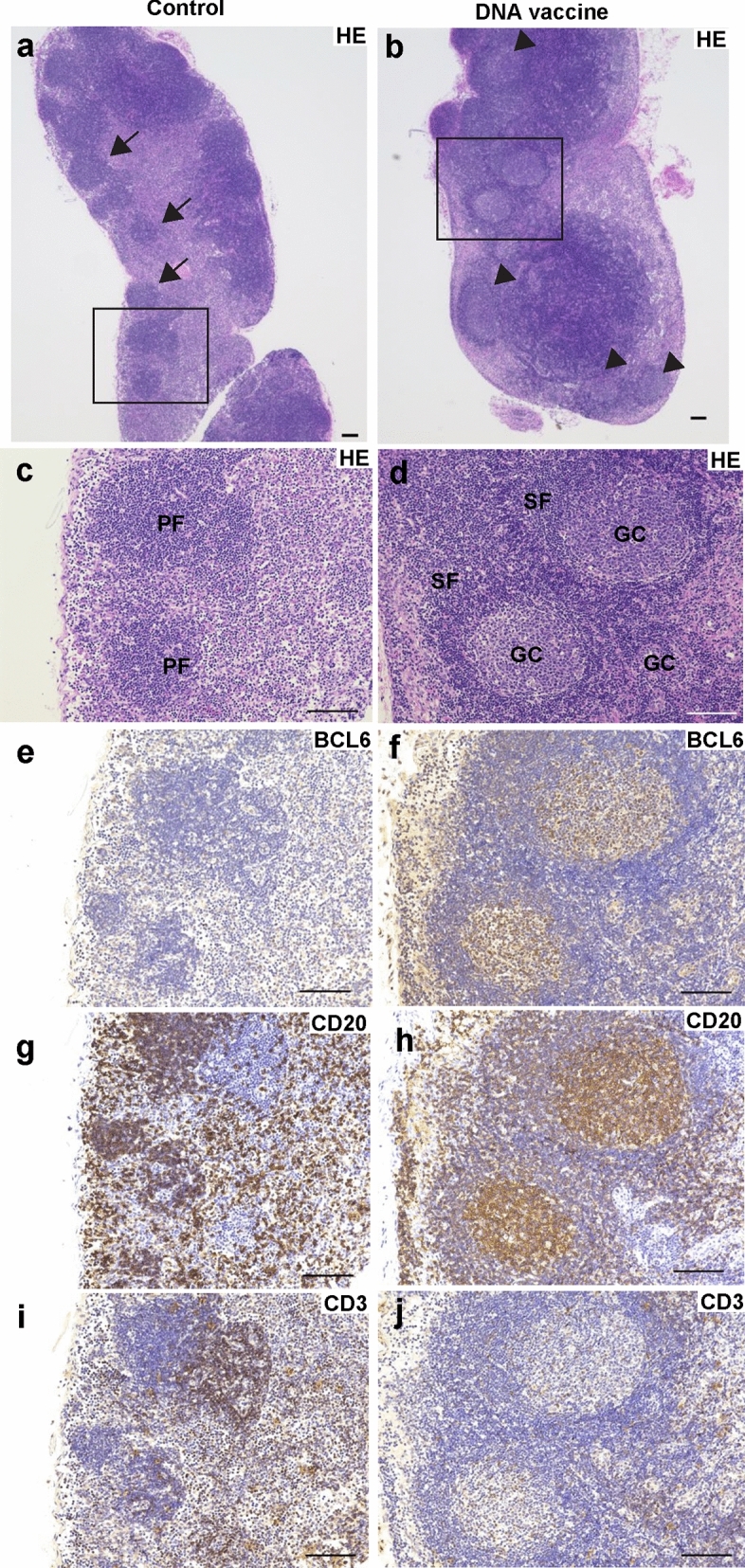
Germinal center formation was detected in lymph nodes after the SARS-CoV-2 Omicron-DNA vaccine via PJI. Representative image of HE staining of lymph nodes from control rat (**a**, **c**, **e**, **g**, **i**) or immunized rat with Omicron DNA vaccine (**b**, **d**, **f**, **h**, **j**). (**c**, **d**) Enlarged image of black square of (**a**, **b**). Arrow indicates primary follicle. Arrow head indicates secondary follicle with germinal center formation. (**e**–**j**) immunohistochemistry with germinal center with BCL6 (**e**, **f**), CD20 (**g**, **h**), and CD3 (**i**, **j**). *PF* primary follicle, *SF* secondary follicle, *GC* germinal center. Scale bar = 100 μm. See also Supplementary Fig. 5.

To produce long-lived antibody from B cells or memory B cells, germinal center formation is known to be critical in peripheral lymphoid organ in infection or vaccination^22–24^. We analyzed the germinal center formation in lymph node by histological analysis. Lymph nodes was collected and stained with hematoxylin and eosin (HE) at 7 weeks after 1st dose of DNA vaccine (at 3 weeks after 3rd dose). In control group, there are only primary follicles. By contrast, many secondary (activated) follicles with germinal center formation in Omicron DNA vaccine group (Fig. 3a–d and Supplementary Fig. 5a–d). By immunohistochemical analysis, cells in germinal centers in DNA vaccine group were stained with BCL6 (follicular B and T cells), CD20 (B cells), and CD3 (T cells) (Fig. 3e–i). These suggested that PJI-injected Omicron DNA vaccine induced germinal center responses, leading to long-lasting antibody production.

### Omicron DNA vaccine elicits cellular immune responses

**Figure 4:**
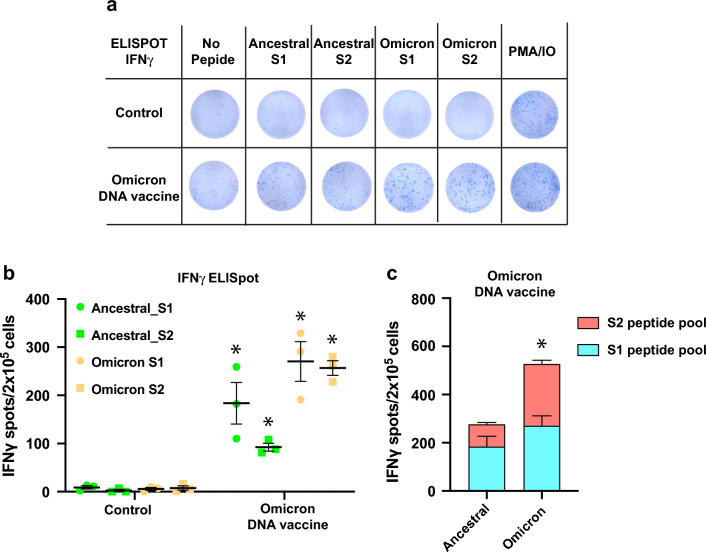
Cellular immune responses induced by SARS-CoV-2 Omicron DNA vaccine. (**a**) Representative image of ELISpot assay. Spike-specific T-cell responses were analyzed by ELISpot assay. At 7 weeks after the first dose of DNA vaccine, the vaccinated or control splenocytes were isolated and stimulated with spike peptide pool from ancestral as well as Omicron BA.1. (**b**) Quantitative analysis of IFNγ-releasing cells from ELISpot assay. The number of spots were analyzed after subtraction from background spots (no peptide stimulation) of each group. *p < 0.05 versus control (**c**) Quantitative data of the total number of spots (S1 + S2) of IFNγ-releasing cells from each strain (S1 and S2 peptide pool). *p < 0.05 versus ancestral spike. N = 3. The experiments were repeated twice. Data are presented as mean ± SEM.

SARS-CoV-2 spike-specific cellular immune responses evoked by the DNA vaccine were monitored via PJI-mediated intradermal administration^20,21^. We evaluated the omicron variant-specific cellular immune responses using the ELISpot assay. The splenocytes responded to S1 and S2 peptide pools from ancestral and Omicron BA.1 to produce IFNγ (Fig. 4a, b). However, the total number of spots (S1 + S2) belonging to ancestral spike protein was lower than those generated by Omicron BA.1 (Fig. 4c). Collectively, this data suggests that the Omicron DNA vaccine could elicit a cellular immune response in all the tested strains; however, it was relatively weaker in case of the ancestral spike peptide pool.

## Discussion

Omicron B.1.1.529-adapted DNA vaccine elicited a humoral immune response, with the BA.1-specific antibody titer being highest at 6 weeks after the first vaccination dose; the response eventually tapered toward 30 weeks. Antibody titers for BA.1 and BA.2 were significantly decreased between 6 and 30 weeks. However, neutralization activity did not decrease even at 30 weeks. In lymph nodes, the affinity of some antibodies against antigens is enhanced by affinity maturation and somatic mutation in the germinal center^25–28^. The generation of memory B cells and long-lived plasma cells for prolonged neutralizing antibody production from antigen-specific germinal center reactions is required for a vaccine to exert its effectivity^29^. Recent studies indicate that approved SARS-CoV-2 mRNA vaccines induce a germinal center reaction in humans and mice^30–33^. In this study, Omicron DNA vaccine via PJI induced secondary (activated) follicles with germinal center formation, which are mainly containing germinal center B cells, stained with BCL6, CD20 (Fig. 3). By contrast, less number of primary follicles, which are mostly composed on naïve B cells, were detected (Fig. 3 and Supplementary Fig. 5)^34^. Taken together, we speculate that Omicron-adapted DNA vaccine developed based on our DNA vaccine platform may induce the prolonged antibody production, and affinity maturation. However, further investigation is needed to evaluate follicular helper T cells, supporting germinal center reaction^35^, and germinal center B cells^24^ induced by DNA vaccine.

Cross-reactive cellular immune response was also activated by the Omicron-adapted DNA vaccine, similar to the original DNA vaccine. However, the Omicron-variant DNA vaccine elicited a relatively weaker activation against the ancestral stain (Fig. 4). Omicron spike-specific T-cell response was significantly decreased in peripheral blood mononuclear cells from individuals who had received ancestral stain-targeted vaccines^36–38^. In the spike protein, 88.4% of T cell epitopes are conserved between the ancestral and Omicron (B.1.1.529) variants^36^, which affects spike-specific T cell responses. In our study, rat splenocytes vaccinated with the Omicron-adapted DNA vaccine exhibited reduced activation against ancestral spike stimulation, especially for S2 peptides pool (Fig. 4). The further analysis of T cell epitope will be necessary to explain these results. However, ancestral spike-specific T cell activation was maintained, suggesting that the Omicron-targeted DNA vaccine might also induce protective T cell immunity against infection by potential re-emerging previous VOC-related variants with highly conserved T cell epitopes^39^.

It is known that intradermal delivery of vaccine is effective than others, due to enriched population of immune cells such as antigen presenting cells (APCs)^40,41^. The advantage of PJI, needle-less injector, using pyro-technology is well-controlled injection by two types adjustable explosives^42^. It was reported that PJI precisely and effectively delivered plasmid DNA into intradermal region, leading to enhanced antigen expression and humoral immune response, compared with a conventional intradermal injection using needle-syringe delivery^20,43^. Our results in this study shows that PJI-mediated DNA vaccine robustly induced germinal center responses (Fig. 3), where activated B cells undergo clonal evolution by somatic mutations, affinity maturations, with the help of follicular helper T cells and follicular dendritic cells to produce more durable and broadly reactive antibodies against SARS-CoV-2 variants^24,44^. Although further investigations are needed to elucidate the effect of PJI on innate immune responses, the efficient intradermal antigen expression induced by PJI confers strong humoral immune responses including germinal center reactions for broadly protection.

The result of a phase I study to evaluate the safety and immunogenicity of intradermal DNA vaccine targeting ancestral strain via pyro-drive jet injector in healthy adults has been reported with unfavorable consequence^45^. In this clinical study, pVAX1-based DNA vaccine targeting ancestral strain was intradermally administrated via PJI into healthy individuals at 2 doses, low-dose (0.2 mg) or high-dose (0.4 mg) twice at 2-weeks interval. While, Zydus cadila, Indian Pharmaceutical company, also developed a pVAX1-based DNA vaccine, SARS-CoV-2 vaccine (ZyCoV-D). Their DNA vaccine received emergency use authorization in India^46^. In their clinical trial, DNA vaccine was administrated at low-dose (1 mg), or high-dose (2 mg) three times (total amount of DNA vaccine: 6 mg (2 mg × 3 times), which lead to humoral and cellular immune responses^47^. Those suggested that the weak humoral response may be because of insufficient antigen expression^45^. The enhancement of antigen expression might be one of ways to induce better humoral response. We have developed DNA vaccine by modified spike (amino acid substitutions at K986/V987P, 19 amino acid deletion at C-terminal), resulting in strengthened antigen expression on cell surface level, which leads to better humoral responses compared with original DNA vaccine that was used in clinical trial^21^. Although the difference in immune system between experimental animals and human needs to be considered, introduction of modification in DNA vaccine to enhance antigen expression might be efficient approach for future DNA vaccine development.

As a limitation of this study, we did not performed live SARS-CoV-2 challenge test to evaluate how maintained neutralization activity of antibodies impact on the pathogenicity of live SARS-CoV-2 including circulating or next variants.

In summary, we designed the Omicron B.1.1.529 specific DNA vaccine based on our improved SARS-CoV-2 spike DNA vaccine platform^21^ and evaluated vaccine-induced humoral and cellular immune responses against ancestral and Omicron sub lineages. Our findings confirmed that the Omicron B.1.1.529 strain-adapted DNA vaccine successfully induced germinal center reaction, leading to high antibody titers, which were cross-reactive and durable for 30 weeks, and elicited pseudo virus neutralization activity.

## Methods

### Synthesis of DNA vaccine targeting Omicron variant

The sequence of the SARS-CoV-2 Omicron variant was modified and inserted into the pVAX1 vector plasmid, as previously described^21^. The mutations of Omicron BA.1 spike (B.1.519) were introduced using gene synthesis (A67V, H69del, V70del, T95I, G142D, V143del, Y144del, Y145del, N211del, L212I, ins214EPE, G339D, S371L, S373P, S375F, K417N, N440K, G446S, S477N, T478K, E484A, Q493R, G496S, Q498R, N501Y, Y505H, T547K, D614G, H655Y, N679K, P681H, N764K, D796Y, N856K, Q954H, N969K, L981F). Sequences of the synthesized plasmids were confirmed by DNA sequencing.

### Cell culture and plasmid DNA transfection

HEK293 cells were maintained in Dulbecco’s Modified Eagle Medium (DMEM; Nacalai Tesque) supplemented with 10% fetal bovine serum (FBS; Nichirei) and penicillin/streptomycin (Nacalai Tesque). The cells were then incubated at 37 °C in a humidified 5% CO_2_ incubator (PHCbi). Plasmid DNA transfection was performed using Lipofectamine 2000 (Invitrogen) according to the manufacturer’s instructions.

### Western blotting

After transfection with plasmid DNA, the cells were washed with phosphate-buffered saline (PBS) and lysed with RIPA buffer supplemented with a protease inhibitor cocktail (Roche). Cell lysates were sonicated and centrifuged at 20,000×*g* for 5 min at 4 °C. The supernatant was collected and stored at − 80 °C until further use. The cell lysate was quantified, and the sample was mixed with Laemmli sample buffer (Bio-Rad) along with β-mercaptoethanol. The boiled samples were separated on a 4–20% gradient gel (Daiichi Gel). The separated samples were then transferred to a methanol-activated polyvinylidene difluoride (PVDF) membrane. The PVDF membranes were blocked with 5% skim milk in PBS containing 0.05% Tween 20 (PBS-T) for 1 h at RT. The membrane was then incubated with primary antibodies (SARS-CoV-2 spike #42172, Cell Signaling Technology, GAPDH MAB374, Millipore) overnight at 4 °C. After the membrane was washed with PBS-T, it was incubated with a secondary antibody tagged with horseradish peroxidase (HRP, GE Healthcare) for 1 h at RT. The membrane was washed and developed using a HRP substrate (Chemi-Lumi One L, Nacalai Tesque). The signals were detected using a ChemiDoc™ Touch imaging system (Bio-Rad).

### Immunocytochemistry

Cells were seeded on glass-bottomed plates (Matsunami Glass). After transfection with the plasmid DNA, the cells were washed with PBS and fixed with 4% paraformaldehyde. Fixed cells were blocked with PBS containing 5% skim milk (Wako) for 1 h at RT. The cells were incubated with primary antibodies for the SARS-CoV-2 Spike (BLSN-005P, Beta Lifescience) or control IgG (Thermo Fisher) at 4 °C overnight. The following day, cells were washed and incubated with a secondary antibody labeled with Alexa Fluor 488 (Molecular Probes) for 1 h at RT. Nuclei were stained with 4′, 6-diamidino-2-phenylindole (DAPI, Roche). The cells were observed under a confocal microscope (FV10i; Olympus).

### Animals and immunization protocol

Female Crl: Caesarean Derived (CD) Sprague Dawley (SD) rats (7 weeks old; Charles River) were housed with free access to food and water in a temperature- and light-cycle-controlled facility. DNA vaccines were intradermally administered using a pyro-drive jet injector (PJI, Actranza Lab, Type: Rat, provided by Daicel. Co.). For the vaccination, 240 μg of DNA vaccine was administered into flank regions of rats by PJI thrice at 2-week intervals (2 mg/mL of DNA vaccine, 30 μL/injection × 4 sites/animal). Control group: N = 3. DNA vaccine group: N = 6. All animal experiments were performed under isoflurane-, or three types of mixed anesthetic agents (medetomidine, midazolam, and butorphanol)-mediated anesthesia. All studies involving animals are reported in accordance with *ARRIVE* guidelines.

### Determination of antibody titers using enzyme-linked immunosorbent assay (ELISA)

Anti-spike antibody titers were measured using ELISA as previously described^19^. Briefly, 96-well plates were coated with 1 μg/ml recombinant spike protein (ancestral spike; SPN-C52H9, Omicron BA.1 spike; SPN-C52Hz, Omicron BA.2 spike; SPN-C5223, Omicron BA.5 spike; SPN-C522e, Acro Biosystems) and kept at 4 ℃ overnight. After blocking with 5% skim milk PBS for 2 h at RT, the diluted sera (ranging from 10- to 31,250-fold dilution with 5% skim milk PBS) were added to the wells and incubated at 4 °C overnight. The following day, the wells were washed with PBS-T and incubated with HRP-conjugated anti-IgG antibody (GE Healthcare) for 3 h at RT. The wells were developed using the peroxidase chromogenic substrate 3,3′-5,5′-tetramethyl benzidine (TMB, Sigma) for 30 min at RT. The reaction was quenched by adding sulfuric acid (0.5 N). The absorbance of the samples in the wells was measured immediately at 450 nm using a microplate reader (Bio-Rad). The half-maximum antibody titer of the sample was determined from the highest absorbance in the dilution range (GraphPad Prism 8 software). For IgG subclass determination, anti-IgG1 (Abcam), IgG2a (Abcam), IgG2b (Abcam), and IgG2c (Southern Biotech) antibodies were used as the secondary antibodies.

### Enzyme-linked Immunospot (ELISpot) assay

SARS-CoV-2 spike-specific cellular immune responses were evaluated by ELISpot assay using an Immunospot® kit (Cellular Technology Limited). Briefly, PVDF membrane-bottomed 96-well plates were activated with methanol and then coated with an anti-rat interferon γ (IFNγ) capture antibody at 4 °C overnight. On the following day, the splenocytes, collected from vaccinated or control rats, were incubated with ancestral or Omicron spike peptide pools (Ancestral; PM-WCPV-S-SU1-1 and SU2-1, Omicron BA.1; PM-SARS2-SMUT08-1, JPT peptide technologies) for 24 h at 37 °C in a humidified CO_2_ incubator. After washing, the anti-IFNγ-detecting antibody was added to the wells and incubated for 2 h at RT. IFNγ spots were developed using the Tertiary solution and Blue developer solution. Finally, the number of IFNγ-spots (IFNγ-releasing splenocytes) was counted using the Immunospot analyzer. The spot values were subtracted from background spots for analysis.

### Pseudo-typed virus production and neutralization assay

Vesicular stomatitis virus (VSV)-based pseudo-typed viruses with a SARS-CoV-2 spike protein (ancestral spike: GenBank: QZC47358.1, Omicron BA.1 spike: UGN73932.1, Omicron BA.2 spike: UF069279.1, Omicron BA.5: UTZ18966.1) were generated.

For the neutralization assay, serially diluted serum samples were incubated with the pseudo-typed virus and added to Vero cells. After 24 h at 37 °C, the cells were lysed and activated using the luciferase assay system (Promega). Luciferase activity was measured using Synergy LC (BioTek). The neutralization value was analyzed using GraphPad Prism 8 based on 50% inhibitory dilution (ID50).

### Histology and immunohistochemistry

For histological analysis, inguinal lymph nodes were collected at 7 weeks after the first vaccination and fixed in 4% paraformaldehyde. Fixed tissues were embedded in paraffin, cut and stained with hematoxylin and eosin (HE).

For immunohistochemical analysis of CD3 (T cells), CD20 (B cells), and Bcl6 (follicular B cells and T cells), the deparaffinized sections were heated for antigen retrieval. After blocking with Antibody Diluent with Background Reducing Components (Dako), the sections were incubated with primary antibodies (CD3: ab16669 abcam, CD20: ab64088 abcam, Bcl6: ab272859 abcam) for overnight at 4 °C. After washing, the sections were treated with HRP-labelled pokymer anti-rabbit (Dako Envision system, Dako) for 30 min at RT, and developed with the ImmPACT DAB system (Vector) for 5 min. The sections were counterstained with Mayer’s hematoxylin. Stained tissue sections were observed under a BZ-X810 microscope (Keyence).

### Statistical analysis

All values are presented as mean ± SEM. To assess significant differences, Student’s *t-*test, as well as one- and two-way ANOVA, were performed. These were followed by Tukey’s or Bonferroni’s multiple comparison test using GraphPad Prism 8 software. Finally, statistical significance was set at *p* < 0.05.

### Statements

All methods in this study were performed in accordance with the relevant guidelines and regulations.

### Ethics approval

All experiments were approved by the Ethical Committee for Animal Experiments of Osaka University Graduate School of Medicine.

### Supplementary Information

Supplementary Information.

## Data Availability

The datasets used in this study are available upon a reasonable request from the corresponding author. The plasmid sequence of pVAX1-SARS-CoV-2 Omicron DNA vaccine have been deposited into DDBJ/EMBL/GenBank under Accession Number LC760234.

## References

[1] Krammer F (2020). SARS-CoV-2 vaccines in development. Nature.

[2] Baden LR (2021). Efficacy and safety of the mRNA-1273 SARS-CoV-2 vaccine. N. Engl. J. Med..

[3] Polack FP (2020). Safety and efficacy of the BNT162b2 mRNA covid-19 vaccine. N. Engl. J. Med..

[4] Voysey M (2021). Safety and efficacy of the ChAdOx1 nCoV-19 vaccine (AZD1222) against SARS-CoV-2: An interim analysis of four randomised controlled trials in Brazil, South Africa, and the UK. Lancet.

[5] Cox M (2022). SARS-CoV-2 variant evasion of monoclonal antibodies based on in vitro studies. Nat. Rev. Microbiol..

[6] Newman J (2022). Neutralizing antibody activity against 21 SARS-CoV-2 variants in older adults vaccinated with BNT162b2. Nat. Microbiol..

[7] Wang L (2022). Differential neutralization and inhibition of SARS-CoV-2 variants by antibodies elicited by COVID-19 mRNA vaccines. Nat. Commun..

[8] Dejnirattisai W (2022). SARS-CoV-2 Omicron-B.1.1.529 leads to widespread escape from neutralizing antibody responses. Cell.

[9] Sharma V, Rai H, Gautam DNS, Prajapati PK, Sharma R (2022). Emerging evidence on Omicron (B.1.1.529) SARS-CoV-2 variant. J. Med. Virol..

[10] VanBlargan LA (2022). An infectious SARS-CoV-2 B.1.1.529 Omicron virus escapes neutralization by therapeutic monoclonal antibodies. Nat. Med..

[11] Chalkias S (2022). A bivalent Omicron-containing booster vaccine against Covid-19. N. Engl. J. Med..

[12] Fang Z (2022). Omicron-specific mRNA vaccination alone and as a heterologous booster against SARS-CoV-2. Nat. Commun..

[13] Wu Y (2022). Omicron-specific mRNA vaccine elicits potent immune responses in mice, hamsters, and nonhuman primates. Cell Res..

[14] Kis Z (2022). Stability modelling of mRNA vaccine quality based on temperature monitoring throughout the distribution chain. Pharmaceutics.

[15] Uddin MN, Roni MA (2021). Challenges of storage and stability of mRNA-based COVID-19 vaccines. Vaccines (Basel).

[16] Kyriakidis NC, Lopez-Cortes A, Gonzalez EV, Grimaldos AB, Prado EO (2021). SARS-CoV-2 vaccines strategies: A comprehensive review of phase 3 candidates. NPJ Vaccines.

[17] Gary EN, Weiner DB (2020). DNA vaccines: Prime time is now. Curr. Opin. Immunol..

[18] Shafaati M (2021). A brief review on DNA vaccines in the era of COVID-19. Future Virol..

[19] Hayashi H (2022). Preclinical study of a DNA vaccine targeting SARS-CoV-2. Curr. Res. Transl. Med..

[20] Nishikawa T (2022). Immune response induced in rodents by anti-CoVid19 plasmid DNA vaccine via pyro-drive jet injector inoculation. Immunol. Med..

[21] Hayashi H (2022). Modified DNA vaccine confers improved humoral immune response and effective virus protection against SARS-CoV-2 delta variant. Sci. Rep..

[22] De Silva NS, Klein U (2015). Dynamics of B cells in germinal centres. Nat. Rev. Immunol..

[23] MacLennan IC (1994). Germinal centers. Annu. Rev. Immunol..

[24] Stebegg M (2018). Regulation of the germinal center response. Front. Immunol..

[25] Kotaki R (2022). SARS-CoV-2 Omicron-neutralizing memory B cells are elicited by two doses of BNT162b2 mRNA vaccine. Sci. Immunol..

[26] Moriyama S (2021). Temporal maturation of neutralizing antibodies in COVID-19 convalescent individuals improves potency and breadth to circulating SARS-CoV-2 variants. Immunity.

[27] Muecksch F (2022). Increased memory B cell potency and breadth after a SARS-CoV-2 mRNA boost. Nature.

[28] Muecksch F (2021). Affinity maturation of SARS-CoV-2 neutralizing antibodies confers potency, breadth, and resilience to viral escape mutations. Immunity.

[29] Sallusto F, Lanzavecchia A, Araki K, Ahmed R (2010). From vaccines to memory and back. Immunity.

[30] Kim W (2022). Germinal centre-driven maturation of B cell response to mRNA vaccination. Nature.

[31] Lederer K (2020). SARS-CoV-2 mRNA vaccines foster potent antigen-specific germinal center responses associated with neutralizing antibody generation. Immunity.

[32] Mudd PA (2022). SARS-CoV-2 mRNA vaccination elicits a robust and persistent T follicular helper cell response in humans. Cell.

[33] Turner JS (2021). SARS-CoV-2 mRNA vaccines induce persistent human germinal centre responses. Nature.

[34] Allen CD, Cyster JG (2008). Follicular dendritic cell networks of primary follicles and germinal centers: Phenotype and function. Semin. Immunol..

[35] Crotty S (2019). T follicular helper cell biology: A decade of discovery and diseases. Immunity.

[36] Choi SJ (2022). T cell epitopes in SARS-CoV-2 proteins are substantially conserved in the Omicron variant. Cell. Mol. Immunol..

[37] Keeton R (2022). T cell responses to SARS-CoV-2 spike cross-recognize Omicron. Nature.

[38] Naranbhai V (2022). T cell reactivity to the SARS-CoV-2 Omicron variant is preserved in most but not all individuals. Cell.

[39] Tarke A (2022). SARS-CoV-2 vaccination induces immunological T cell memory able to cross-recognize variants from Alpha to Omicron. Cell.

[40] Hickling JK (2011). Intradermal delivery of vaccines: Potential benefits and current challenges. Bull. World Health Organ..

[41] Kim YC, Jarrahian C, Zehrung D, Mitragotri S, Prausnitz MR (2012). Delivery systems for intradermal vaccination. Curr. Top Microbiol. Immunol..

[42] Miyazaki H, Atobe S, Suzuki T, Iga H, Terai K (2019). Development of pyro-drive jet injector with controllable jet pressure. J. Pharm. Sci..

[43] Chang C (2019). Stable immune response induced by intradermal DNA vaccination by a novel needleless pyro-drive jet injector. AAPS PharmSciTech.

[44] Laidlaw BJ, Ellebedy AH (2022). The germinal centre B cell response to SARS-CoV-2. Nat. Rev. Immunol..

[45] Nakagami H (2022). Phase I study to assess the safety and immunogenicity of an intradermal COVID-19 DNA vaccine administered using a pyro-drive jet injector in healthy adults. Vaccines (Basel).

[46] Sheridan C (2021). First COVID-19 DNA vaccine approved, others in hot pursuit. Nat. Biotechnol..

[47] Momin T (2021). Safety and immunogenicity of a DNA SARS-CoV-2 vaccine (ZyCoV-D): Results of an open-label, non-randomized phase I part of phase I/II clinical study by intradermal route in healthy subjects in India. EClinicalMedicine.

