# Supplementary material for: Intradermal administration of DNA vaccine targeting Omicron SARS-CoV-2 via pyro-drive jet injector provides the prolonged neutralizing antibody production via germinal center reaction
Source: Sci Rep. 2023 Aug 10;13:13033. doi: 10.1038/s41598-023-40172-y (PMC10415318; doi:10.1038/s41598-023-40172-y)
Supplement: Supplementary file 1 — Supplementary Information. [file 41598_2023_40172_MOESM1_ESM.pdf]

Supplementary Figure 1.

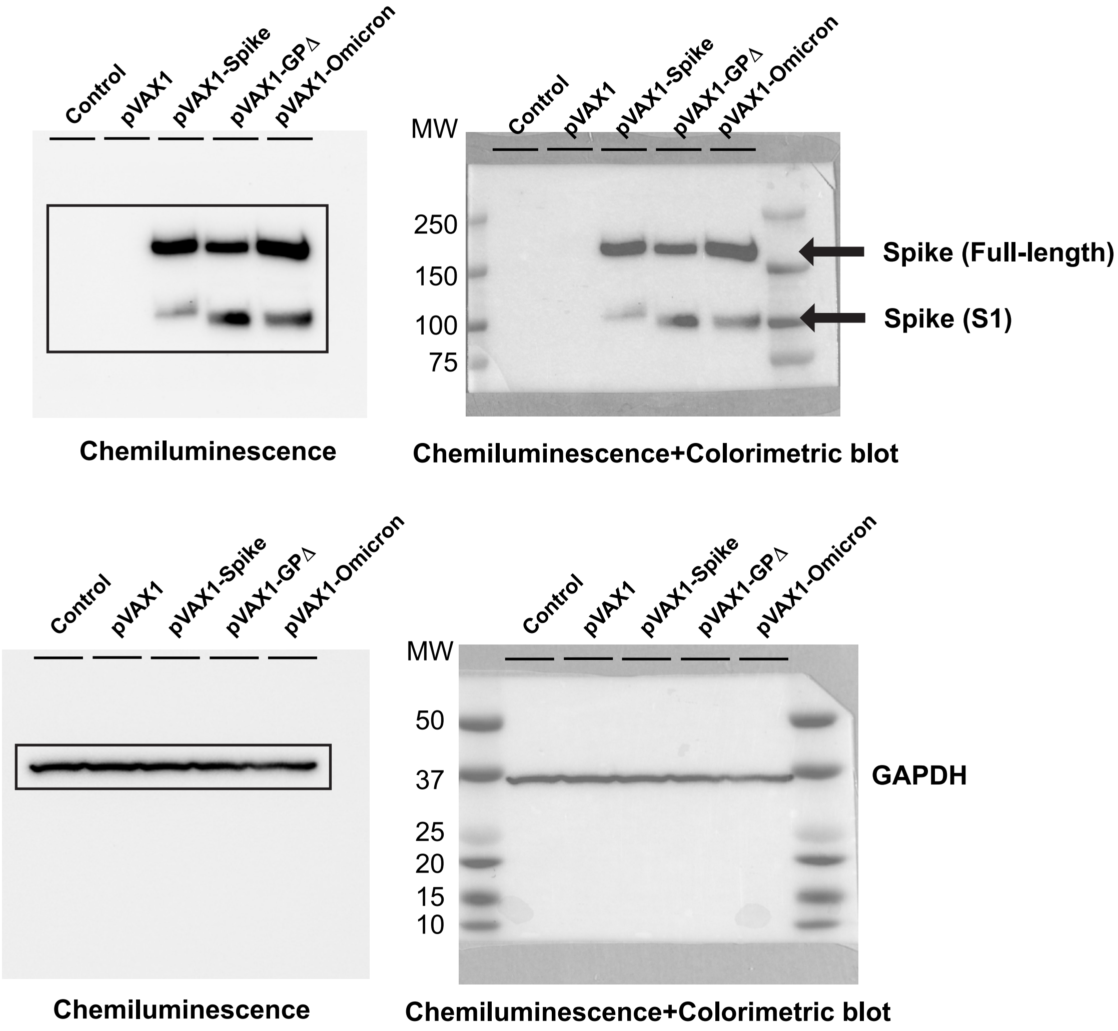

Supplementary Figure 1. Uncropped western blots related to Figure 1b.
Uncropped full-length images (chemiluminescence with or without clolorimetric image) evaluating spike and GAPDH expression are shown. The cropped images are shown in Figure 1b.

Supplementary Figure 2.

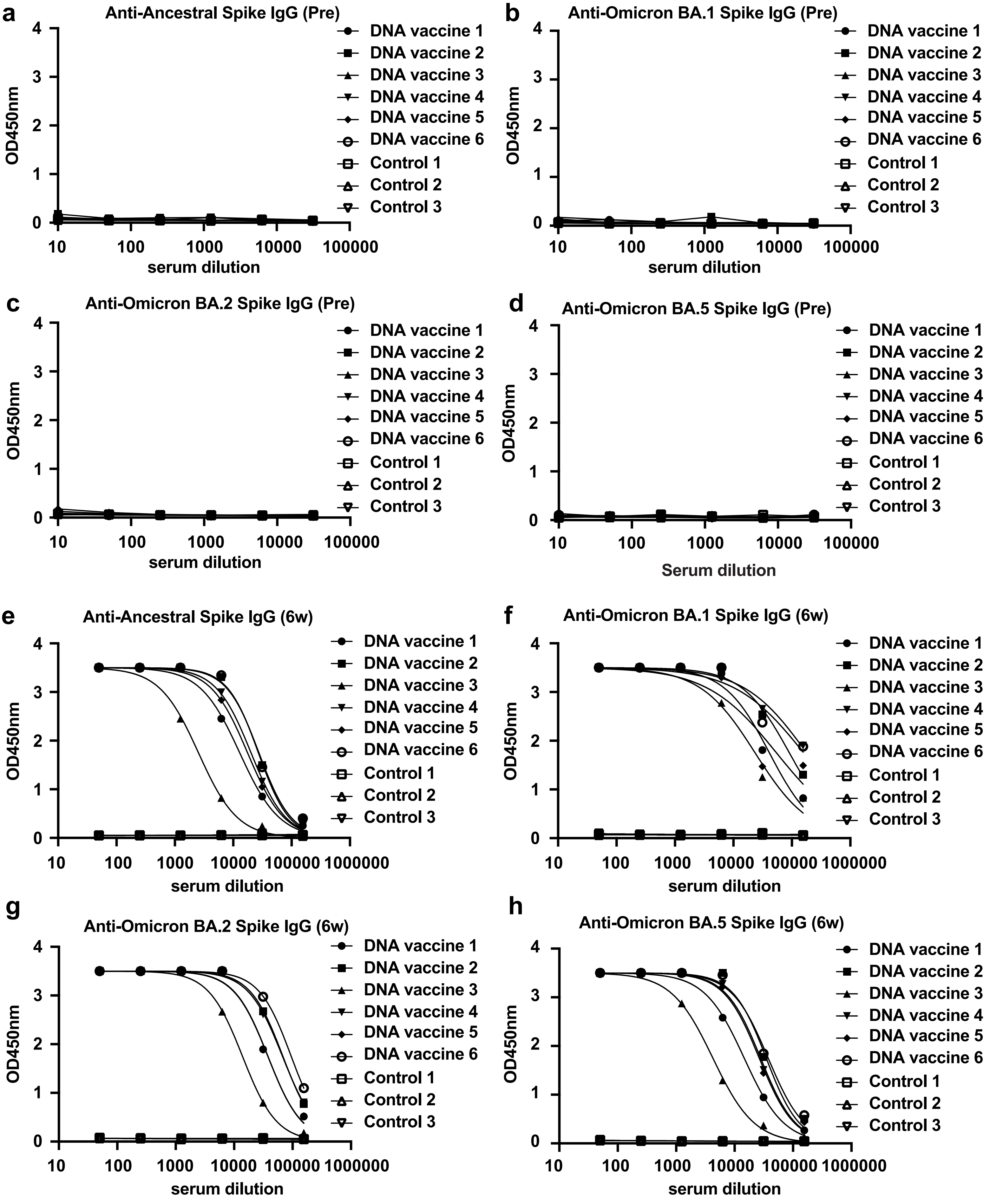

Supplementary Figure 2. Humoral immune responses by Omicron DNA intradermal vaccine at pre-vaccination and 6 w
(a-d) Antibody titers for recombinant ancestral **(a)**, Omicron BA.1 **(b)**, Omicron BA.2 **(c)** and Omicron BA.5 **(d)** before vaccination. **(e-h)** Antibody titers for recombinant ancestral **(e)**, Omicron BA.1 **(f)**, Omicron BA.2 **(g)** and Omicron BA.5 **(h)** at 6 w after 1st dose of Omicron DNA vaccine.

Supplementary Figure 3.

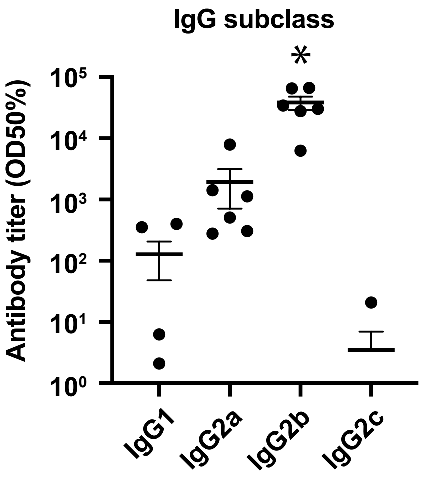

Supplementary Figure 3. Spike-specific IgG subclass induced by Omicron DNA vaccine
IgG subclass for omicron BA.1-spike was measured by ELISA. * $p<0.01$ vs. IgG1, IgG2a, and IgG2c, respectively. N=6.

Supplementary Figure 4.

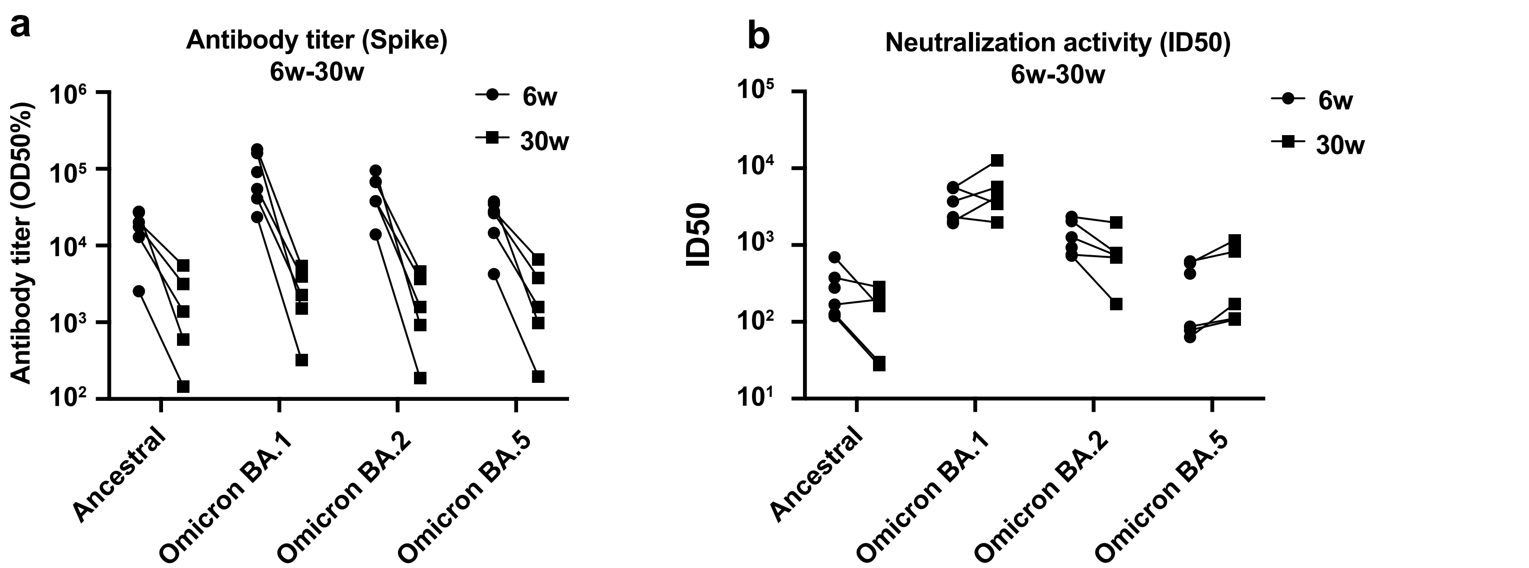

Supplementary Figure 4. Individual data of spike-specific IgG antibody titer and neutralization activity
(a) Individual data of antibody titers for ancestral, omicron BA.1, BA.2, and BA.5 spike at 6 w and 30 w after 1st dose of Omicron DNA vaccine. **(b)** Individual data of neutralization activity, measured by pseudo-virus assay with ancestral, omicron BA.1, BA.2, and BA.5 spike at 6w and 30 w after 1st dose of Omicron DNA vaccine. N=5-6.

Supplementary Figure 5.

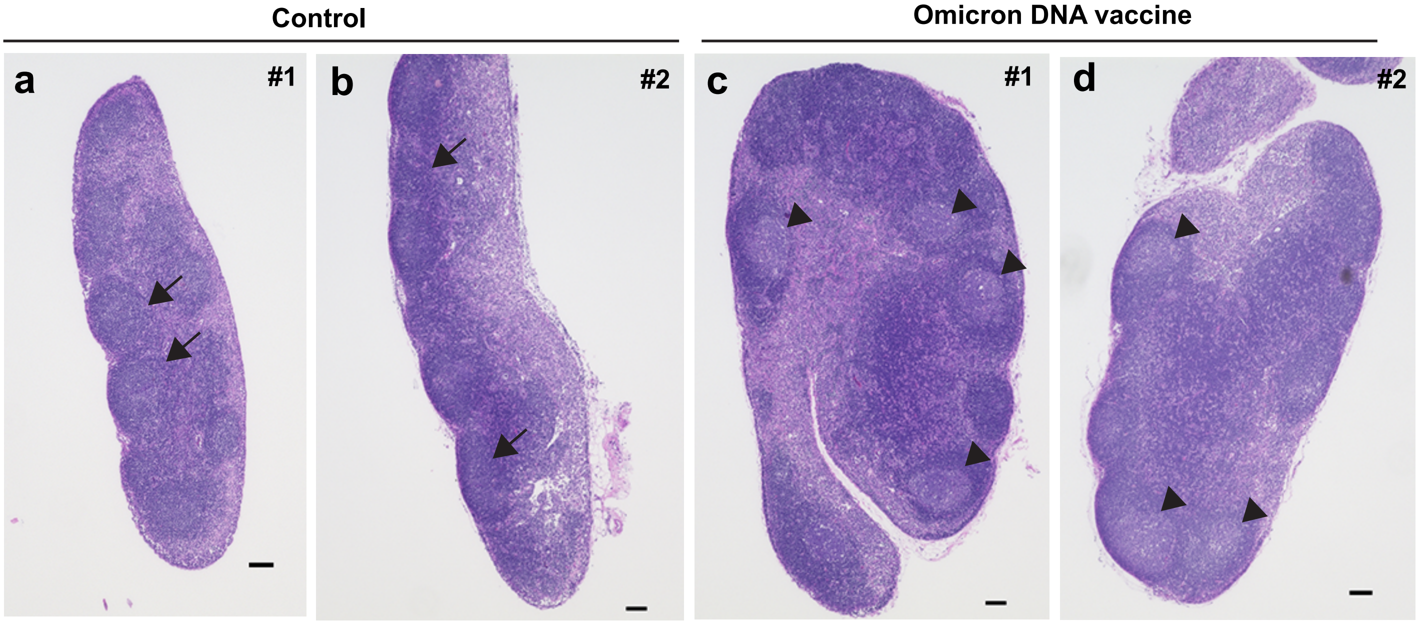

Supplementary Figure 5. Detection of secondary follicles with germinal center formation by Omicron DNA vaccine in lymph nodes. Lymph nodes from **(a-b)** control 2 rats (#1, #2) or **(c-d)** immunized 2 rats (#1, #2) with Omicron DNA vaccine were stained with HE at 7 weeks. Arrow indicates primary follicle. Arrow head indicates secondary follicle with germinal center formation. Scale bar = 100 μm.
